# Review: Roles of Prebiotics in Intestinal Ecosystem of Broilers

**DOI:** 10.3389/fvets.2018.00245

**Published:** 2018-10-30

**Authors:** Po-Yun Teng, Woo Kyun Kim

**Affiliations:** Department of Poultry Science, University of Georgia, Athens, GA, United States

**Keywords:** prebiotic, broilers, immunity, microbiota, mannan oligosaccharides, β-glucans, fructans

## Abstract

In recent years, prebiotics have been considered as potential alternatives to antibiotics. Mechanisms by which prebiotics modulate the ecosystem of the gut include alternation of the intestinal microbiota, improvement of the epithelium, and stimulation of the immune system. It is suggested that the administration of prebiotics not only influences these aspects but also regulates the interaction between the host and the intestinal microbiota comprehensively. In this review, we will discuss how each prebiotic ameliorates the ecosystem by direct or indirect mechanisms. Emphasis will be placed on the effects of prebiotics, including mannan oligosaccharides, β-glucans, and fructans, on the interaction between the intestinal microbiota, gut integrity, and the immunity of broilers. We will highlight how the prebiotics modulate microbial community and regulate production of cytokines and antibodies, improving gut development and the overall broiler health. Understanding the cross talk between prebiotics and the intestinal ecosystem may provide us with novel insights and strategies for preventing pathogen invasion and improving health and productivity of broilers. However, further studies need to be conducted to identify the appropriate dosages and better resources of prebiotics for refinement of administration, as well as to elucidate the unknown mechanisms of action.

## Introduction

Since the use of antibiotic growth promoters was banned by the EU on January 1st, 2006, several feed additives have been studied as alternatives to antibiotics, such as probiotics, prebiotics, synbiotics, and herbal medicines (1). Among these feed additives, prebiotics have been studied and supplemented broadly into broiler diets in recent years. Gibson and Roberfroid (2) defined a prebiotic compound as a non-digestible food ingredient utilized by intestinal microbiota. It beneficially affects the host by selectively stimulating the growth and/or activity of one or a limited number of bacteria in the intestinal tract, consequently improving gut health and hosts' intestinal microbial balance. Gibson et al. (3) revised the definition and defined a prebiotic as a selectively fermented ingredient that allows specific changes in the composition and/or activity in the intestinal microbiota that confers benefits upon the host's well-being and health. Some researchers also confined prebiotics to indigestible oligosaccharides (4). Ideal characteristics of prebiotics were described by Patterson and Burkholder (5): (1) prebiotics should not be hydrolyzed by animal gastrointestinal enzymes, (2) prebiotics cannot be absorbed directly by cells in the gastrointestinal tracks, (3) prebiotics selectively enrich one or limited numbers of beneficial bacteria, (4) prebiotics alter the intestinal microbiota and their activities, and (5) prebiotics ameliorate luminal or systemic immunity against pathogen invasion.

The ecosystem of the gut is composed of three crucial elements: (1) microbial community, (2) intestinal epithelial cells, and (3) immune system (6). Generally, prebiotics can be fermented by health-promoting bacteria in the intestine, producing lactic acid, short-chain fatty acid (SCFA), or some antibacterial substances, such as bacteriocine against pathogenic species (7). These products may not only benefit the intestinal microbial structure but also improve the integrity of intestinal epithelial cells, which further increase the absorption of nutrients and enhance the growth performance of animals (8).

Intestinal microbiota are influenced by various factors, including diet, gender, background genotype, housing environment, litter, and also age of birds (9). These factors can alter the abundance of dominant bacterial phyla and families in each part of the intestine. For instance, gut microbiota in young chickens changed rapidly with increase of age. *Clostridiaeae* and *Enterobacteriaceae* are two dominant families in the ileum of 7 day-old chickens, whereas *Lactobacillaceae* and *Clostridiacea* represent the common families in the ileum of 35 day-old birds (9). However, the balance of intestinal microbiota is alterable. Application of prebiotics in diets could establish a healthy microbial community in the intestine of young broilers by enhancing the abundance of *Lactobacilli* and *Bifidobacteria* and reducing the titers of *Coliform* (10, 11).

Furthermore, the modulation of intestinal microbiota is associated with immune responses. On the one hand, inhibiting pathogen colonization by prebiotics can decrease detrimental molecules produced by pathogenic bacteria, which have been known as exogenous signals (12). These signals are also called pathogen-associated molecular patterns (PAMPs). The PAMPs can be recognized by pattern recognition receptors (PRR), including toll-like receptors (TLRs) and NOD-like receptors (NLRs), which are expressed on the surface of sentinel cells (13). Once PRRs recognize PAMPs, sentinel cells, such as epithelial cells, macrophages, mast cells, and dendritic cells, are activated, producing cytokines for the regulation of further innate immune responses. On the other hand, prebiotics can act as non-pathogenic antigens themselves. They can be recognized by receptors of immune cells, which consequently modulate host immunity beneficially.

**Figure 1:**
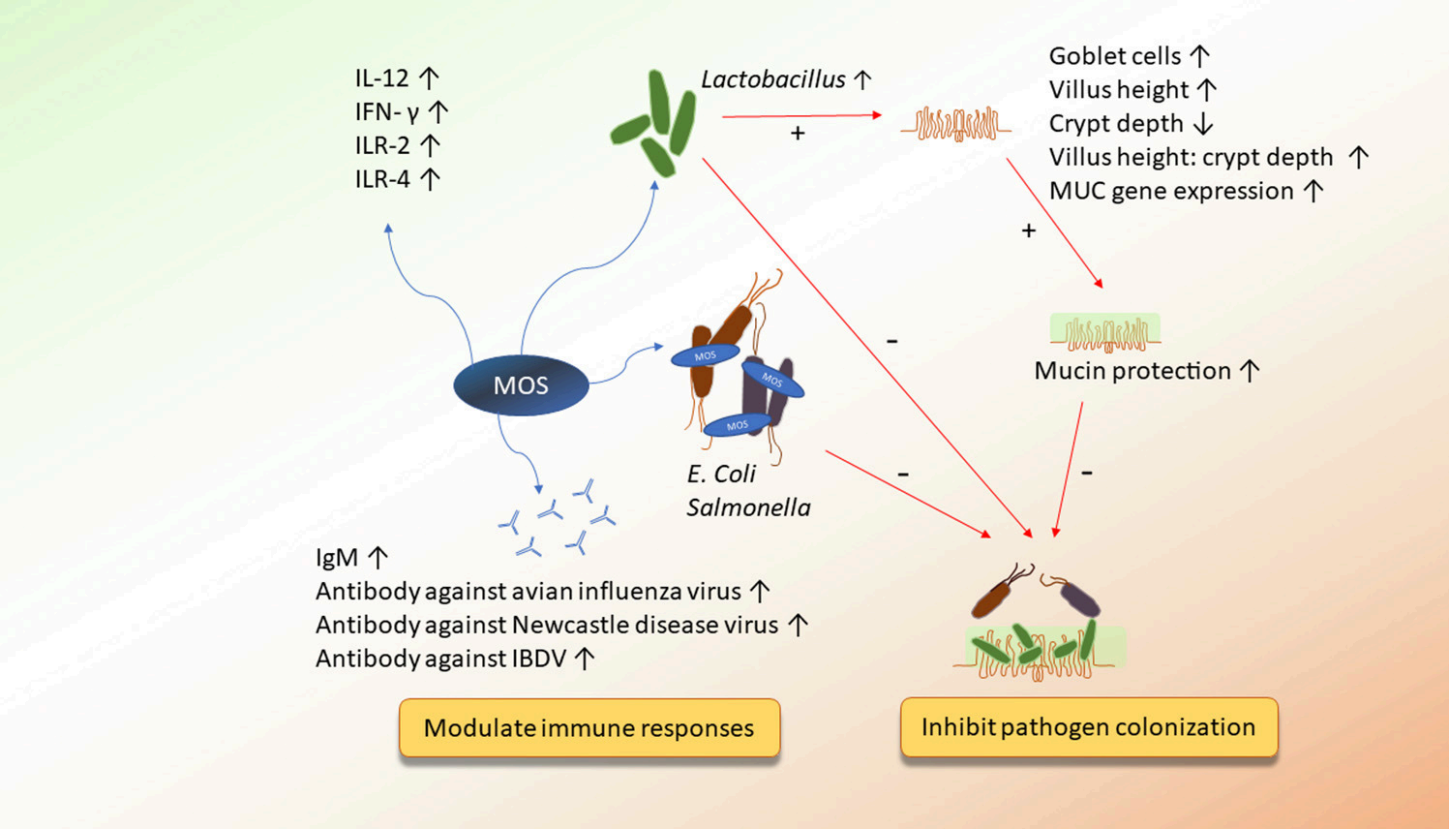
The potential mechanisms of action of MOS on improving immunity and inhibiting pathogen colonization.

**Figure 2:**
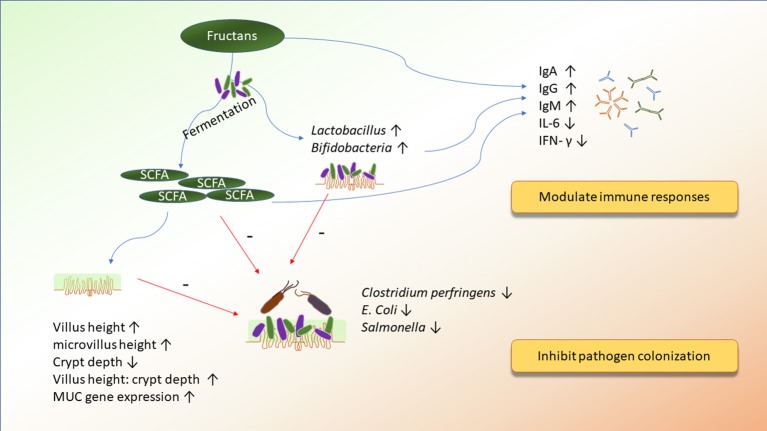
The potential mechanisms of action of fructans on improving immunity and inhibiting pathogen colonization.

**Figure 3:**
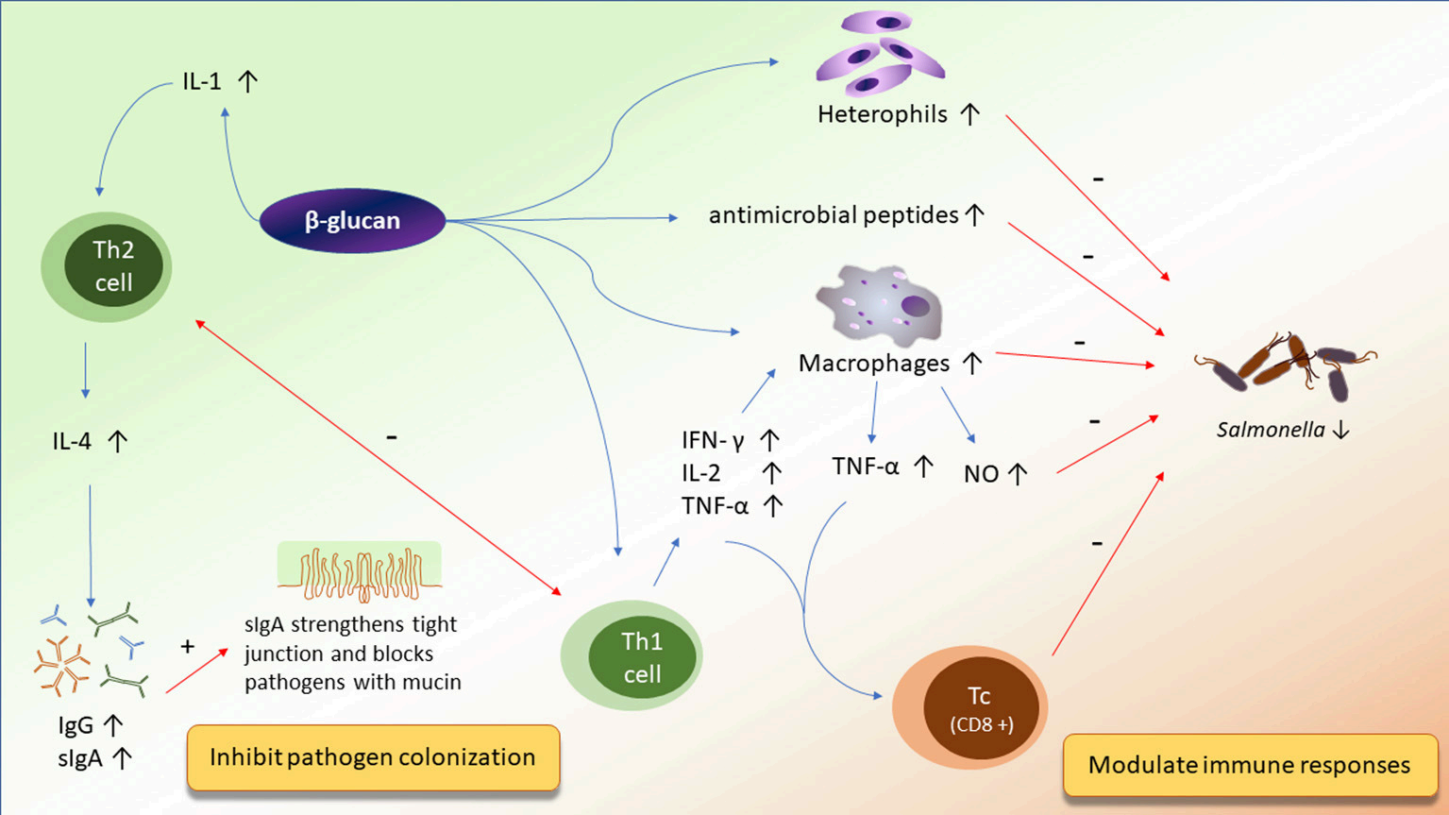
The potential mechanisms of action of β-glucan on improving immunity and inhibiting pathogen colonization.

Various prebiotics are composed of diverse sugar units. Therefore, each prebiotic may influence the animals differently. Here, we reviewed studies of broilers that discuss the effects of prebiotics on their underlying mechanisms of action. We will discuss the direct or indirect mechanisms by which prebiotics ameliorated the ecosystem of the chicken gut. Emphasis will be placed on the impacts of mannan oligosaccharides, β-glucans, and fructans on the interaction between the intestinal microbiota, immunity, and the integrity of the epithelial cells (Figures 1–3).

## Mannan oligosaccharides (MOS)

Most of the mannan oligosaccharide (MOS) products are derived from yeast cell walls (*Saccharomyces cerevisiae*) and are rich in mannoproteins (12.5%), mannan (30%), and glucan (30%) (14, 15). Mannan oligosaccharides are known for their ability to bind pathogenic bacteria, which possess type-1 fimbriae, such as *E. coli* and *Salmonella* species (16). By blocking bacterial lectin, MOS could reduce colonization of these pathogens in the intestine of animals (17). Previous studies indicated that supplementation of MOS from 0.08 to 0.5% could alter cecal microbial community composition by increasing total anaerobic bacteria, *Lactobacillus* and *Bifidobacterium*, and decreasing *Salmonella, E. coli, Clostridium perfringens*, and *Campylobacter* (14, 16, 18–23). Apart from its effects on cecal microbiota, MOS also improved microbial community in other sections of the intestine, including the jejunum, the ileum, the jejunal mucosa, the ileal mucosa, and the ileocecal junction (11, 22, 24–26). It is interesting to note that MOS increased cecal *Bacteroidetes* in 7 and 35 day-old broilers (23, 27). Genus *Bacteroides* have been known for their strong metabolic activity. They can efficiently ferment indigestible polysaccharides to SCFA and, consequently, improve nutrient absorption and protect the host from pathogen infection (28). In previous studies, shown in Table 1, *Lactobacillus* species were the main species influenced by MOS. Mannan oligosaccharides increased the prevalence of ileal *L. acetotolerans, L. delbrueckii* subsp. *lactis, L. sakei* subsp. *sakei*. and cecal *L. ingluviei, L. mucosae, L. salivarius*, and *L. crispatus* (23, 29). Among these *Lactobacillus* species, *L. crispatus* was reported to have anti-*E. coli* and anti-*Salmonella* activities, whereas *L. salivarius* was mentioned to have the ability to limit *Salmonella* colonization (30, 31). The anti-pathogenic characteristics of *Lactobacillus* may be the reason why MOS reduced the numbers of *E. coli* or *Salmonella* in the intestine, ameliorating bacterial infection in pathogen-challenged broilers (14, 16, 19).

**Table 1:** Effects of mannan oligosaccharides on intestinal microbiota of broilers.

**Effects**		**Dosage, Challenge, and Diets**	**Day**	**References**
**Jejunum**				
Alter	Community composition	0.5%	25	(24)
**Jejunal mucosa**				
Decrease	*Coliforms*	0.2% with *E. coli* challenge	7	(10)
**Ileum**				
Increase	Calculated Sorenson's similarity indices (Cs)/ intragroup	0.2%	21	(11)
Increase	Total anaerobic bacteria	0.2%	7	(10)
Decrease	*Coliforms*	0.2%	7	(11)
Decrease	*Coliforms*	0.2%	14	(10)
Decrease	*Clostridium perfringens*	0.2%	21	(11)
Increase	Diversity of *Lactobacillus*	0.2%	21	(11)
Increase	*Lactobacillus*	0.2%	7	(11)
Decrease	*Lactobacillus*	0.2%	14	(22)
Increase	*Lactobacillus*	0.017% MOS and 0.025% β-glucan	14	(26)
Increase	*L. acetotolerans*	0.2%	21	(11)
Increase	*L. delbrueckii* subsp. *lactis*	0.2%	21	(11)
Increase	*L. sakei* subsp. *Sakei*	0.2%	21	(11)
**Ileal mucosa**				
Increase	*Lactobacillus*	0.2%	21	(11)
Increase	*L. acetotolerans*	0.2%	21	(11)
**Ileocecal junction**				
Decrease	*Clostridium perfringens*	0.1%	28	(25)
Decrease	*E. coli*	0.1%	28	(25)
Decrease	*Lactobacillus*	0.1%	28	(25)
**Ceca**				
Alter	Community composition	0.08% in starter and 0.04% in finisher	7, 35	(23)
Alter	Community composition	0.1%	28	(29)
Alter	Community composition	0.2%	14, 28	(29)
Alter	Community composition	0.5%	25	(24)
Increase	Total anaerobic bacteria	0.2%	7	(11)
Decrease	*Firmicutes*	0.08% in starter and 0.04% in finisher	35	(23)
Decrease	*Coliforms*	0.2% in wheat diet	21	(21)
Decrease	*Salmonella*	0.4% with *Salmonella dublin* Challenge	10	(16)
Decrease	*Salmonella*	0.4% with *Salmonell typhimurium* Challenge	10	(16)
Decrease	*E. coli*	0.2% in starter and 0.1% in finisher with *E. coli* challenge	9	(19)
Decrease	*E. coli*	0.2% in starter and 0.1% in finisher	3, 28, 42	(19)
Decrease	*E. coli*	0.2% or 0.5%	34	(14)
Decrease	*Clostridium perfringens*	0.2%	21	(10)
Decrease	*Clostridium perfringens*	0.4% in wheat diet	21	(20)
Decrease	*Campylobacter*	0.2% in Dextrose-ISP diet	34	(14)
Increase	*Kitastosphora*	0.20%	21	(11)
Increase	*Bacteroides*	0.08% in starter and 0.04% in finisher	7, 35	(23)
Increase	*Bacteroides*	0.20%	7	(27)
Increase	*Bifidobacteria*	0.20%	34	(14)
Increase	*Bifidobacteria*	0.5% (MOS and β-glucan)	14, 24, 34	(14)
Decrease	*Lactobacillus*	0.10%	14	(22)
Increase	*Lactobacillus*	0.20%	24	(14)
Increase	*Lactobacillus*	0.50%	34	(14)
Increase	*Lactobacillus*	0.2% in starter and 0.1% in finisher	38, 42	(19)
Increase	*L. ingluviei*	0.20%	21	(11)
Increase	*L. mucosae*	0.20%	21	(11)

In addition, higher levels of intestinal *Lactobacillus* in birds fed with MOS may further result in the improvement of gut health status. Mannan oligosaccharides have been reported to increase villus height and surface area, decrease crypt depth, induce numbers of sulphated-acidic goblet cells, and upregulate gene expression of *MUC*, which is related to mucin secretion (10, 11, 14, 32–35) (Table 2). It has been reported that sulphated-acidic goblet cells are less degradable by the pathogen's glycosides (43, 44). Therefore, they can provide stronger protection against pathogens for the host. Similarly, Cheled-Shoval et al. (36) reported that *in ovo* administration of MOS enhanced villus area and proliferation of goblet cells. The greater numbers of goblet cells were able to increase the gene expression of *MUC*, synthesizing and secreting more mucin, which plays an important role as the first line of defense. Mucin can trap pathogens or impede them from invading epithelial cells (45). Thus, it is hypothesized that MOS establishes a bidirectional interaction: the increase of *Lactobacillus* counts may improve intestinal development, whereas mucin produced by goblet cells can conversely limit attachment of pathogens to epithelial cells.

**Table 2:** Effects of prebiotics on intestinal morphology of broilers.

**Effects**		**Dosage, Challenge, and Diets**	**Day**	**References**
**MOS**				
**Intestine**				
Increase	MUC2 gene expression	0.1% 0.6 ml *in ovo*	E20 (embryonic)	(36)
**Duodenum**				
Increase	Goblet cell numbers	0.2%	34	(14)
Increase	Villus height	0.5%	14	(14)
Increase	Villus height	0.2%	34	(14)
Increase	Villus height	0.2% with *Salmonella typhimurium* challenge	10	(35)
Increase	Villus height: crypt depth	0.2% with *Salmonella typhimurium* challenge	10	(35)
Increase	Villus surface area	0.2% with *Salmonella typhimurium* challenge	10	(35)
**Jejunum**				
Increase	Goblet cell numbers	0.2%	24, 34	(14)
Increase	Goblet cell numbers	0.5%	24, 34	(14)
Increase	Goblet cell numbers	0.1%	16, 26	(34)
Increase	Villus height	0.2%	24	(14)
Increase	Villus height	0.1%	26	(34)
Increase	Villus height	0.2% with *Salmonella typhimurium* challenge	10	(35)
Increase	Villus height: crypt depth	0.2% with *Salmonella typhimurium* challenge	10	(35)
Increase	Villus surface area	0.2% with *Salmonella typhimurium* challenge	10	(35)
Decrease	Crypt depth	0.2%	7	(10)
Decrease	Crypt depth	0.2% in wheat diet	7	(21)
**Ileum**				
Increase	Goblet cell numbers	0.2%	24	(14)
Increase	Goblet cell numbers	0.1%	16, 26	(34)
Increase	Villus height	0.1%	26	(34)
Increase	Villus height	0.2%	21	(11)
Increase	Villus height	0.2% with *Salmonella typhimurium* challenge	10	(35)
Increase	Cup area	0.2%	21	(11)
Increase	Goblet cell density (acidic)	0.2%	21	(11)
Increase	Goblet cell density (sulphated-acidic)	0.2%	21	(11)
Increase	Goblet cell density (total)	0.2%	21	(11)
Decrease	Goblet cell density (sialo-acidic)	0.2%	21	(11)
β**-glucan**				
**Jejunum**				
Increase	Villus height	0.01% with *Salmonella typhimurium* challenge	21	(37)
	Villus height: crypt depth	0.01% with *Salmonella typhimurium* challenge	21	(37)
	Goblet cell density	0.01% with *Salmonella typhimurium* challenge	21	(37)
Decrease	MUC2 gene expression	0.1% with *Eimeria* challenge	14	(38)
Increase	MUC2 gene expression	0.1%	14	(38)
Increase	MUC2 gene expression	0.1% with *Eimeria* challenge	21	(38)
Increase	Claudin-1	0.01% with *Salmonella typhimurium* challenge	21	(37)
Increase	Occludin	0.01% with *Salmonella typhimurium* challenge	21	(37)
**Fructan**				
**Jejunum**				
Increase	MUC gene expression	1%	21, 42	(39)
Increase	MUC gene expression	1.5%	21	(39)
Increase	Microvillus height	0.4%	49	(40)
Decrease	Crypt depth	0.4%	49	(40)
Increase	Villus height: crypt depth	0.4%	49	(40)
**Ileum**				
Increase	Villi height	0.4%	49	(40)
Increase	Microvillus height	0.2%	49	(40)
Increase	Microvillus height	0.4%	49	(40)
Decrease	Crypt depth	0.4%	49	(40)
Increase	Villus height: crypt depth	0.2%	49	(40)
Increase	villus height: crypt depth	0.4%	49	(40)
**Ceca**				
Increase	Villus height: crypt depth	0.1%	35	(41)
**XOS**				
**Ileum**				
Increase	Villus height	0.5%	26	(42)
**GGMO**				
**Duodenum**				
Increase	Villus height	0.1, 0.2, or 0.3% with *Salmonella typhimurium* challenge	10	(35)
Increase	Villus height: crypt depth	0.1, 0.2, or 0.3% with *Salmonella typhimurium* challenge	10	(35)
Increase	Villus surface area	0.1, 0.2, or 0.3% with *Salmonella typhimurium* challenge	10	(35)
**Jejunum**				
Increase	Villus height	0.1, 0.2, or 0.3% with *Salmonella typhimurium* challenge	10	(35)
Increase	Villus height: crypt depth	0.1, 0.2, or 0.3% with *Salmonella typhimurium* challenge	10	(35)
Increase	Villus surface area	0.1, 0.2, or 0.3% with *Salmonella typhimurium* challenge	10	(35)
**Ileum**				
Increase	Villus height	0.1, 0.2, or 0.3% with *Salmonella typhimurium* challenge	10	(35)
Increase	Villus height: crypt depth	0.1, 0.2, or 0.3% with *Salmonella typhimurium* challenge	10	(35)
Increase	Villus surface area	0.1, 0.2, or 0.3% with *Salmonella typhimurium* challenge	10	(35)

The effects of MOS on immunity of broilers are presented in Table 3. TLR4 and TLR2 were upregulated in the ileum or cecal tonsils by 0.2% MOS supplementation (50). It indicated that MOS could be recognized by both TLR4 and TLR2. Similar to mammalian TLR4, chicken TLR4 (chTLR4) mRNA has been found in a wide range of cells, particularly in macrophages and heterophils (61). TLR4 is a receptor that recognizes lipopolysaccharide (LPS) in mammals. After recognizing LPS, immune cells could produce high levels of nitric oxide and pro-inflammatory cytokines against pathogenic bacteria. Thus, it was suggested that reducing the exposure of LPS from *E. coli* by MOS could downregulate gene expression of chTLR4 and inhibit pro-inflammatory immunity (50). However, molecules of MOS can be recognized by TLR4 as well. It was reported that MOS may act as a pro-inflammatory factor that upregulates TLR4 gene expression and induces innate immune responses (62).

**Table 3:** Effects of mannan oligosaccharides, β-glucan, and fructans on immune responses of broilers.

**Effects**		**Dosage, Challenge, and Diets**	**Day**	**References**
**MOS**				
**Blood/Serum**				
Decrease	B cell	0.5%	25	(46)
Increase	IgM	0.5%	25	(46)
Increase	Antibody against Avian Influenza virus	0.1, 0.2, 0.3% with ND vaccination	42	(47)
Increase	Antibody against Avian Influenza virus	0.1, 0.2, 0.3%	42	(47)
Increase	Antibody against Newcastle disease virus	0.09%	42	(48)
Increase	Antibody against IBDV	0.5%	54 weeks	(49)
Increase	Antibody against sheep red blood cell	0.09%	28, 42	(48)
Increase	Total antibody against sheep red blood cell	0.09%	28, 42	(48)
Decrease	Basophils	0.2%	28	(25)
Decrease	Heterophil: lymphocyte	0.2%	28	(25)
**Ileum**				
Increase	IFN-γ	0.2%	22	(50)
Increase	IFN-γ	0.2% with *Clostridium perfringens* challenge	22	(50)
Increase	IL-12p35	0.2%	22	(50)
Increase	IL-12p35	0.2% with *Clostridium perfringens* challenge	22	(50)
Increase	TLR2b	0.2%	22	(50)
Increase	TLR2b	0.2% with *Clostridium perfringens* challenge	22	(50)
Increase	TLR4	0.2%	22	(50)
Increase	TLR4	0.2% with *Clostridium perfringens* challenge	22	(50)
Decrease	TLR2	0.1% 0.6 ml *in ovo*	1, 3	(36)
Increase	TLR4	0.1% 0.6 ml *in ovo*	1	(36)
**Cecal tonsils**				
Decrease	B cell	0.5%	25	(46)
Increase	IFN-γ	0.2%	22	(50)
Increase	IFN-γ	0.2% with *Clostridium perfringens* challenge	22	(50)
Decrease	TLR2b	0.2% with *Clostridium perfringens* challenge	22	(50)
Increase	TLR4	0.2%	22	(50)
Increase	TLR4	0.2% with *Clostridium perfringens* challenge	22	(50)
**MOS and** β**-glucan**				
**Blood/Serum**				
Increase	Antibody/ infectious bursal virus	0.1% with *Salmonella* enteritidis challenge	21, 42	(51)
Decrease	Eosinophils	0.1% with *Salmonella* enteritidis challenge	42	(51)
Increase	Monocytes	0.1% with *Salmonella* enteritidis challenge	42	(51)
β**-glucan**				
**Abdominal exudate cell macrophages**				
Increase	Nitrite	1, 2.5, 5 mg/ml	35	(52)
Increase	Phagocytic activity	0.002, 0.004%	35	(52)
Increase	lL-1	5 mg/ml	35	(52)
Increase	Total antibody responses to Sheep red blood cell	0.004%	35	(52)
**Intraepithelial leukocytes**				
Increase	CD4+	0.004%	16	(52)
Increase	CD8+	0.004%	16	(52)
**MQ-NCSU**				
Increase	Nitrite	1, 5 mg/ml	35	(52)
Increase	Macrophages	5 mg/ml	35	(52)
**Organ**				
Increase	Bursa weight %	0.002, 0.004%	14	(52)
Increase	Spleen weight %	0.002, 0.004%	14	(52)
Decrease	Liver *Salmonella* enteritidis invasion	with *Salmonella* enteritidis challenge	4	(53)
Decrease	Spleen *Salmonella* enteritidis invasion	with *Salmonella* enteritidis challenge	4	(53)
**Intestine**				
Increase	sIgA	0.0025, 0.005, 0.0075, 0.01, 0.0125%	21, 42	(54)
Decrease	IL-4	0.10%	21	(38)
**Intestinal fluid**				
Decrease	IgG	0.0001%	7, 28	(55)
**Duodenum**				
Decrease	IFN-γ	0.1% with *Eimeria* challenge	10	(38)
Decrease	IFN-γ	0.1%	7	(56)
Decrease	IL-4	0.02, 0.1%	7	(56)
Increase	IL-4	0.02, 0.1%	14	(56)
Decrease	IL-8	0.02, 0.1%	7, 14	(56)
Decrease	IL-13	0.1%	7	(56)
Decrease	IL-18	0.02%	14	(56)
Increase	Nitic oxide synthase	0.1%	14	(56)
**Jejunum**				
Decrease	IFN-γ	0.1%	14	(38)
Decrease	IFN-γ	0.1% with *Eimeria* challenge	14	(38)
Decrease	IFN-γ	0.1%	7	(56)
Decrease	IL-4	0.1%	7	(56)
Decrease	IL-8	0.1%	7	(56)
Decrease	IL-8	0.02%	14	(56)
Decrease	IL-13	0.1%	7	(56)
Decrease	IL-18	0.1%	14	(56)
Increase	IL-18	0.1%	21	(38)
Increase	IL-18	0.02%	7	(56)
Increase	*Cath-1*	0.02%	14	(57)
Increase	*Cath-2*	0.02%	14	(57)
Increase	*AvBD-1*	0.02%	22	(57)
Increase	*AvBD-4*	0.02%	22	(57)
Increase	*AvBD-10*	0.02%	22	(57)
Decrease	*AvBD-10*	0.02% with *Salmonella* enteritidis challenge	22	(57)
Increase	*LEAP-2*	0.02%	22	(57)
Decrease	Nitric oxide synthase	0.1% with *Eimeria* challenge	10	(38)
Increase	sIgA+ cell numbers	0.01% with *Salmonella typhimurium* challenge	21	(37)
Increase	sIgA	0.01% with *Salmonella typhimurium* challenge	14, 21	(37)
Increase	IgA against *Salmonella*	0.02%	22	(57)
**Ileum**				
Decrease	IFN-γ	0.1%	21	(38)
Decrease	IFN-γ	0.1% with *Eimeria* challenge	21	(38)
Decrease	IFN-γ	0.1%	7	(56)
Decrease	IL-4	0.1%	7	(56)
Increase	IL-4	0.1%	14	(56)
Decrease	IL-8	0.1%	7, 14	(56)
Decrease	IL-8	0.02%	14	(56)
Decrease	IL-13	0.1%	7	(56)
Decrease	nitric oxide synthase	0.1%	14	(38)
Increase	nitric oxide synthase	0.1% with *Eimeria* challenge	14	(38)
Increase	nitric oxide synthase	0.1%	14	(56)
**Blood/Serum**				
Increase	Globulin	0.0025, 0.005, 0.0075, 0.01, 0.0125%	21	(54)
Increase	Globulin	0.0025, 0.005, 0.0075, 0.01%	42	(54)
Increase	IFN-γ	0.005, 0.0075%	21	(54)
Increase	IFN-γ	0.01%	42	(54)
Increase	IgG	0.0025, 0.005, 0.0075, 0.01%	21	(54)
Increase	IgG	0.0025, 0.005, 0.0075%	42	(54)
Increase	IgG against *Salmonella*	0.02% with *Salmonella* enteritidis	14, 22	(57)
Increase	IL-1	0.0025, 0.005%	42	(54)
Increase	IL-1	0.01%	21	(54)
Increase	IL-2	0.0025, 0.005, 0.0075, 0.01, 0.0125%	21, 42	(54)
Increase	TNF-α	0.005, 0.0075, 0.01%	21, 42	(54)
Decrease	lymphocytes	0.012% and exposed to LPS	42	(58)
Decrease	lymphocytes	0.05%, and exposed to pokeweed mitogen	42	(58)
Increase	mean number of SE per heterophil	with *Salmonella* enteritidis challenge	4	(53)
Increase	percent heterophils containing SE	with *Salmonella* enteritidis challenge	4	(53)
Increase	phagocytic index	with *Salmonella* enteritidis challenge	4	(53)
Increase	SE Killing/heterophils	with *Salmonella* enteritidis challenge	4	(53)
Increase	nitric oxide/3, 6, 12 h	0.025%, and exposed to LPS	42	(58)
**Fructans**				
**Blood/Serum**				
Decrease	B cells	0.5%	25	(46)
Increase	IgG	0.5%	25	(46)
Increase	IgM	0.5%	25	(46)
Increase	Antibody against sheep red blood cells in primary response	0.05%	42	(59)
**Ileum**				
Increase	CD4+:CD8+	0.5%	21	(39)
Decrease	IFN-γ	0.5%	21	(39)
Decrease	IFN-γ	1%	21	(39)
Increase	IgA	1%	21, 42	(39)
Increase	IgA	1.5%	21	(39)
Increase	IgA	0.5%	42	(39)
Decrease	IL-6	0.5%	21	(39)
**Cecal tonsils**				
Decrease	CD80	0.2 ml (1.76 mg) *in ovo*	35	(60)
Decrease	IFN-B	0.2 ml (1.76 mg) *in ovo*	35	(60)
Decrease	IL-12p40	0.2 ml (1.76 mg) *in ovo*	35	(60)
Decrease	IL-18	0.2 ml (1.76 mg) *in ovo*	35	(60)
Decrease	IL-4	0.2 ml (1.76 mg) *in ovo*	35	(60)
Decrease	Proliferative competence of *ex vivo* leukocytes	0.5%	25	(46)
Decrease	B cells	0.5%	25	(46)

However, chicken TLR2 (chTLR2) has approximately 50% amino acid identity to mammal TLR2, which can recognize a broad variety of PAMPs, including lipoproteins, aribinomannan, and peptidoglycan fugal zymosan (61). TLR2 may recognize MOS as well, which leads to the pro-inflammatory cytokines' cascade (63). A previous study demonstrated that supplementation of 0.2% MOS in broiler diets enhances ileal gene expression of interleukin-12 (IL-12) and interferon-γ (IFN-γ) (50). Interleukin-12 is a cytokine that stimulates T-helper type-1 cells (Th1 cells) and triggers IFN-γ to induce proliferation and cytotoxicity of immune cells, such as T cells, natural killer (NK) cells, and macrophages (12). Apart from the upregulation of innate immunity, MOS can impact humoral immune responses by acting as adjuvant of vaccines to enhance antibody titers. Previous studies have shown that MOS can strengthen antibody titers against sheep red blood cells, infectious bursal disease virus, Newcastle disease virus, and avian influenza virus (47–49). On the contrary, some reports have noted that antibody titers against Newcastle disease virus and infectious bursal disease virus failed to increase in chickens with MOS supplementation (64, 65). This discrepancy among studies may be based on whether or not broilers are infected with pathogens or the variations in MOS sources and environmental conditions (51).

The effects of MOS on intestinal microbiota have been reported broadly. Most of the MOS additions can significantly improve microbial community composition. However, there has been limited research on the impacts of MOS on mechanisms of immune responses in broilers. Although previous studies have found some auspicious results, further research is necessary to determine further antibody titers and gene expression of TLR or cytokines in order to elucidate how MOS improves the broiler's immunity.

## β-glucan

β-glucan is a prebiotic derived from yeast or fungal cell walls. This long-chain polysaccharide is composed of D-glucose monomers with linkages of β-glycosidic 1-3 bonds, and its side-chains are linked by the 1–6 bonds. β-glucan can be recognized by receptors on sentinel cells, triggering production of cytokines and proliferation of lymphocytes (66). Lymphocytes are classified into three major types. The first type is NK cells, which play an important role in innate immunity. The second type is T cells, which regulate adaptive immunity. The third type is B cells, which produce antibodies against antigens. All types of lymphocytes can be modulated by β-glucan. The influences on immune responses of broilers are shown in Table 3.

Macrophages may be one of the sentinel cells that recognize β-glucan in the animal intestine. When macrophages are activated by β-glucan, they produce inducible nitric oxide synthase (iNOS) (56), an enzyme that produces large amounts of nitric oxide. Reacting with superoxide anion, nitric oxide is oxidized to a highly-toxic nitrogen dioxide radical that can kill a wide range of invading pathogens directly or block their DNA synthesis (12, 52, 58). Moreover, β-glucan exposure also triggers macrophage proliferation, enhances macrophage phagocytic ability, and induces macrophage-modulating gene expression of interleukin-1 (IL-1), interleukin-18 (IL-18), and tumor necrosis factor-α (TNF-α) (38, 52). Increasing TNF-α in birds fed with β-glucan may stimulate the incidence of CD8+ lymphocyte, a receptor expressed only on the cytotoxic T cell (Tc) (52, 54). Thus, it is hypothesized that β-glucans can regulate innate immune response by inducing proliferation of Tc cells to attack pathogen-infected cells.

Heterophils, recruited by sentinel cells, are the major granulocytes in most birds and work in a manner similar to neutrophils in mammals. Lowry et al. (53) showed the increases of heterophil phagocytosis in broilers fed with β-glucan, including enhancing the percentage of heterophils containing *Salmonella enterica*, mean numbers of *Salmonella enterica* per heterophil, and phagocytic index. One reasonable explanation that has been proposed is that the dectin-1 receptor involved in β-glucan recognition on the surface of macrophages may also be present on the surface of heterophils (67). Furthermore, heterophils stimulated by β-glucan can release nitric oxide and kill *Salmonella enterica*, resulting in the reduction of pathogenic organ invasion (53). Apart from heterophils, β-glucan receptors are also present on NK cells in humans (68). Therefore, activating NK cells by β-glucan may be another way to improve immune responses in broilers. On the contrary, Cox et al. (56) indicated that β-glucan could be an anti-inflammatory immunomodulator inhibiting interleukin-8 (IL-8) gene expression. Interleukin-8 is a cytokine produced by macrophages, which can recruit heterophiles to phagocytose pathogens at the site of inflammation (12). The inconsistent results may be attributed to whether or not the birds were challenged by pathogens. In a pathogen-challenging situation, pro-inflammatory immune responses may be enhanced by β-glucan supplementation, whereas in normal circumstances, β-glucan may be an anti-inflammatory modulator.

It was reported that the inclusion of β-glucan in diets could regulate the gene expression of antimicrobial peptides (AMPs) (57). Cathelicidins (Cath), avian β-defensins (AvBDs), and liver-expressed antimicrobial peptides (LEAP) are three major families of AMPs, which are expressed by the lung, intestine, immune, and reproductive organs in chickens (57). Antimicrobial peptides can penetrate the membrane of fungi or bacteria, leading to the death of pathogens. Among AMPs, Cath-1 and Cath-2 proteins have been shown to posses the capacity to bind to LPS, inhibiting LPS-mediated pro-inflammatory immune responses (61). On the other hand, AvBDs expressed in heterophils and the mucosal surface of the intestinal and respiratory tracts can damage pathogens, like *Staphyloccocus aureas, E. coli, Candida albicans, S*. Enteritidis*, S. Typhimurium, Listeria monocytogenes*, and *Campylobacter jejuni* (61). Shao et al. (57) reported that the gene expression of Cath-1, Cath-2, AvBD-1, AvBD-2, AvBD-4, AvBD-6, AvBD-9, and LEAP-2 were increased in *Salmonella-*challenged broilers with β-glucan addition. On the contrary, the same study showed that β-glucan reduced Cath-1, AvBD-4, and AvBD-9 in the spleen of birds without pathogen challenge. It could be concluded that if broilers were under pathogen infection, β-glucan would exhibit a strong protection against *Salmonella* and other pathogens in broilers.

After recognizing β-glucan, sentinel cells secrete cytokines that activate Th1 or Th2 cells. The Th1 cells drive the type-1 pathway attack against intracellular pathogens, whereas Th2 cells dominate the type-2 pathway triggering humoral immunity to upregulate antibody production (69). Although Th1 and Th2 cells could release cytokines to cross-inhibit each other, type-1 and type-2 pathways could both be triggered by β-glucan. In type-1 pathways, interleukin-12 (IL-12), produced by macrophages, is a key cytokine that enhances the proliferation of Th1 cells and the production of IFN-γ (12). Interferon-γ further reinforces with IL-18 in order to trigger the activation of Th1 cells and produce additional IFN-γ and IL-2 for the activation of NK cells, stimulation of macrophages and Tc cells, and inhibition against Th2 cells (12). Previous studies reported that β-glucan upregulates the gene expression of IL-2, IL-18, and IFN-γ (52, 54). Additionally, levels of the cytokines interleukin-4 (IL-4) and interleukin-13 involved in type-2 cell pathways are downregulated by β-glucan as well (56). These outcomes support the hypothesis that β-glucan can stimulate the type-1 pathway and inhibit the type-2 pathway.

However, gene expression of IL-1 involved in the type-2 pathway could also be induced by β-glucan (52). Increasing IL-1 found in abdominal exudate cell macrophages can activate Th2 cells and switch on the type-2 pathway. Once activated, Th2 cells release other cytokines to initiate the subsequent anti-inflammatory immune responses. For instance, IL-4 can suppress Th1 cells' activation, stimulate B cells' growth and differentiation, and activate mast cells to produce immunoglobulins (12). Owing to the suppression of Th1 cells, gene expression of IFN-γ was downregulated in duodenum, jejunum, and ileumthe duodenum, the jejunum, and the ileum by β-glucan in *Eimeria*-challenged broilers (38). On the other hand, enhancing immunoglobulins, including IgG and sIgA, in broilers were found by Zhang et al. (54). This is evidence showing that the type-2 pathway can be upregulated by β-glucan. Shao et al. (57) also reported that anti-*Salmonella* specific IgA levels in the jejunum and anti-*Salmonella* specific IgG levels in the serum were increased in birds fed with β-glucan. Similarly, Shao et al. (37) demonstrated that β-glucan could protect intestinal barrier function in *Salmonella*-challenged birds by increasing the amount of goblet cells and IgA-secreting cells, which enhance the sIgA production. sIgA is an important immunoglobulin that serves as the first line of defense (70). There are three major mechanisms of sIgA to protect the integrity of gut lining from pathogenic invasion (71). Firstly, sIgA interacts with non-pathogenic bacteria and epithelium, which consequently strengthens the tight junctions between intestinal epithelial cells and inhibits nuclear translocation of NF-κB (70). A previous study also confirmed that β-glucan enhanced the production of sIgA to ameliorate the damage of tight junction in the jejunum caused by *Salmonella* (37). Secondly, immune complexes that interact with sIgA are involved in the downregulation of gene expression of pro-inflammatory cytokines that include IFN-γ, TNF-α, and interleukin-6 (IL-6) (70). Thirdly, sIgA blocks pathogens within mucin, selecting and maintaining a favorable balance of microbiota in the intestine (70). Shao et al. (37) showed that increased sIgA by β-glucan was associated with the reduction of cecal *Salmonella* colonization and liver invasion.

In summary, β-glucan affects the broiler's immunity via either the type-1 or the type-2 pathway. The conflicting results among different studies may be attributed to the different dosages offered, different ages of the birds used, different parts of the tissue examined, or numerous resources of the β-glucan supplemented. Inconsistent results have also been demonstrated in other animals. For example, cytokines involved in the type-2 pathway of immune responses were downregulated by β-glucan in humans (72) but upregulated in mice (73). Therefore, additional investigation is needed to understand fully the effects of β-glucan on immune responses of broilers.

## Fructans

Fructans, commonly extracted from different plants, hydrolyzed from polysaccharides, or produced by microorganism, have been administered recently in broiler diets. Fructans are classified into three distinct types: the inulin group, the levan group, and the branched group. Firstly, the inulin group, also known as fructooligosaccharides (FOS) can be divided into different categories based on degrees of polymerization (DP): Inulin, normally extracted from chicory roots (*Cichorium intybus L*.), consists of a DP of 3 to 60, and Oligofructose (OF), which can be generated by partial hydrolysis of inulin, enzymatic conversion of sucrose, or lactose, contains a DP of 2 to 10 (74, 75). Most of the inulin group can be found in plants, which comprise oligosaccharides with β-2,1 fructosyl-fructose linkage with a glucose terminal unit. Secondly, the levan group is another group of fructans, which are mostly linked by β-2,6 fructosyl-fructose bonds. Lastly, fructans, which belong to the branched group, contain both β-2,1 fructosyl-fructose and β-2,6 fructosyl-fructose bonds in fair amounts (76). It is the β-glycosidic bond in fructans that resists their breakdown by digestive enzymes in poultry and enhances the population of beneficial bacteria, such as *Bifidobacteria* and *Lactobacilli*, and suppresses levels of pathogenic bacteria, such as *Clostridium pefringens* and *E. coli*, in the intestine of broilers (25, 40, 77).

Saminathan et al. (78) evaluated the utilization of different oligosaccharides by 11 *Lactobacillus* species isolated from the gastrointestinal tract of chickens. This *in vitro* report showed that FOS were utilized by *Lactobacillus* more efficiently than MOS. The high availability of FOS may be associated with specific enzymatic activity and the oligosaccharide transport system of *Lactobacillus* species (79, 80). However, the intestinal microbiota of a broiler is far more complex than those in *in vitro* trials. The prebiotics may be fermented not only by *Lactobacillus* species but also by other microorganisms in the gastrointestinal tracts of animals. Thus, it cannot be assured that the utilization of FOS and MOS in *in vitro* trials is as efficient as in *in vivo* studies.

In addition, the more DP increased, the more residual FOS remained after fermentation by *Bifidobacteria* (81). A previous study indicated that almost 55 *Bifidobacteria* preferred to grow on short-chain FOS rather than long-chain FOS (75). *Bifidobacteria* could also ferment short-chain FOS to produce more acetic acid and lactic acid compared with long-chain FOS within 24 h (81). Similarly, Perrin et al. (82) reported that the population of *Bifidobacteria* and *Lactobacilli* increased earlier in fecal cultures containing OF instead of inulin. However, an increase in the production of formic acid, acetic acid, and lactic acid and a decrease in numbers of *E. coli* group and *Cluster I clostridia* were both observed in cultures containing OF or inulin after 24-h fermentation (82). The same research group also pointed out that butyric acid might be the major product in the inulin group, whereas more acetic acid and lactate acid could be produced from OF (75).

Long-chain fructans, which are degraded slowly in the animal gut, can pass through the small intestine and be fermented in the distal regions of the intestine. Therefore, the inulin group with higher DP might not affect the microbiota in the jejunum significantly (83), but, instead it might alter microbial structure and increase the concentration of SCFA or lactic acid in the ceca of broilers. Effects of FOS on intestinal microbiota are shown in Table 4. Park et al. (85) demonstrated that FOS increased the Shannon diversity of intestinal microbiome compared with the control treatment. Moreover, similar to *in vitro* results, *Bifidobacteria* and *Lactobacillus* are two major beneficial bacteria that were increased in broilers and hens fed with fructans (40, 41, 76, 84, 88). *Bifidobacteria* and *Lactobacillus* not only produced extracellular enzymes to degrade FOS but also competed with other species of intestinal microorganisms and suppressed the growth of pathogenic bacteria (75). For instance, *Campylobacter* titers in the ceca and large intestine were decreased in broilers fed with FOS (84). Regardless of the supplementation of long-chain FOS or short-chain FOS, a reduction in titers of *C. perfringens* was observed in the ileocecal junction or ceca of broilers (20, 25, 76). Similarly, colonization of cecal *C. perfringens* and *Salmonella typhimurium* was decreased by FOS or FOS combined with competitive exclusion products in *E. coli* or *Salmonella- Typhimurium-*challenged birds, respectively (10, 87). Additionally, diets containing different concentrations of FOS (from 0.25 to 1%) could decrease cecal *E. coli* and *Salmonella* in broilers (25, 40, 76, 84, 86). Besides the prevention of *Salmonella* colonization in the ceca of broilers, previous reports also demonstrated that FOS-supplemented diets decreased ovary, liver, and cecal *Salmonella enteritidis* in laying hens (89, 90). The reduction of these pathogenic bacteria might be attributed to cecal SCFA and lactic acid. Same as *in vitro* results, the concentration of cecal butyric acid and lactic acid was significantly higher in broilers fed with inulin (41, 83). Donalson et al. (89) also showed that 0.75 or 0.375% of FOS combined with alfalfa molt diets could increase the concentration of cecal isobutyric acid in hens. Short-chain fatty acids are important fuels in the intestine, and butyrate is the major one that is metabolized by epithelial cells, providing energy for the growth of mucosal epithelium (91). It is suggested that higher concentrations of butyric acid are associated with the improvement of mucosal structure. Previous studies reported that microvillus height in the jejunum and ileum and the ratio of villus to crypt depth in the ceca were increased by FOS (40, 41). Bogucka et al. (92) also reported that *in ovo* injection of inulin increased villus height in broilers at the first day after hatching. In addition, the use of inulin could increase jejunal mucin mRNA expression to produce more mucin, protecting intestinal epithelial cells in broilers (39). By improving intestinal morphology, FOS could further enhance activities of protease and amylase and nutrient absorption, leading to better growth performance (40).

**Table 4:** Effects of fructans on intestinal microbiota of broilers.

**Effects**		**Dosage, Challenge, and Diets**	**Day**	**References**
**Gizzard**				
Decrease	*Lactobacillus*	1% inulin/female	42	(84)
Increase	*Lactobacillus*	1% oligofructose/male	42	(84)
Increase	*Lactobacillus*	1% oligofructose/female	42	(84)
Increase	*Salmonella*	1% oligofructose/male	42	(84)
Increase	*E. coli*	1% inulin/femlae	42	(84)
**Small intestine**				
Increase	*Bifidobacteria*	0.40%	49	(40)
Increase	*Lactobacillus*	0.40%	49	(40)
Increase	*Lactobacillus*	1% oligofructose/female	42	(84)
Decrease	*E. coli*	1% inulin/female	42	(84)
Increase	*E. coli*	0.40%	49	(40)
**Ileum**				
Increase	Diversity	0.25%	28	(25)
Increase	*Lactobacillus*	0.20%	35	(41)
Increase	Total anaerobic bacteria	1.00%	7	(10)
Decrease	*Coliforms*	1.00%	7	(10)
**Ileocecal junction**				
Increase	*Lactobacillus*	0.25%	28	(25)
Decrease	*Clostridium perfringens*	0.50%	28	(25)
Decrease	*E. coli*	0.25, 0.5%	28	(25)
**Ceca**				
Increase	Shannon diversity	0.1%	42	(85)
Increase	*Alistipes* genus	0.1%	42	(85)
Increase	*Bifidobacteria*	0.40%	49	(40)
Increase	*Bifidobacteria*	0.1, 0.2%	35	(41)
Increase	*Bifidobacteria*	0.25 and 0.5%	31	(76)
Decrease	*Lactobacillus*	0.30%	21, 42	(86)
Increase	*Lactobacillus*	0.2, 0.4%	49	(40)
Increase	*Lactobacillus*	0.25 and 0.5%	31	(76)
Increase	*Lactobacillus*	1% inulin/female	42	(84)
Increase	*Lactobacillus*	1% oligofructose/female	42	(84)
Increase	*Lactobacillus intestinali*	0.1%	14, 28	(85)
Increase	*Faecalibacterium prausnitzii*	0.1%	42	(85)
Decrease	Total anaerobic bacteria	0.30%	42	(86)
Increase	Total anaerobic bacteria	0.40%	49	(40)
Increase	Total anaerobic bacteria	1% inulin/female	42	(84)
Increase	Total anaerobic bacteria	1% oligofructose/female	42	(84)
Decrease	*Campylobacter*	1% oligofructose/male	42	(84)
Decrease	*Campylobacter*	1% oligofructose / male	42	(84)
Decrease	*Clostridium perfringens*	1% with *E. coli* challenge	7	(10)
Decrease	*Clostridium perfringens*	0.4% short chain FOS in dextrose-ISP diet	21	(20)
Decrease	*Clostridium perfringens*	0.25 and 0.5%	31	(76)
Decrease	*Coli bacillus*	0.30%	42	(86)
Decrease	*Salmonella*	1% inulin/female	42	(84)
Decrease	*Salmonella*	1% oligofructose/female	42	(84)
Decrease	*Salmonella Typhimurium*	1% and defined competitive exclusion with *Salmonella typhimurium* challenge	7	(87)
Decrease	*E. coli*	0.2, 0.4%	49	(40)
Decrease	*E. coli*	0.25 and 0.5%	31	(76)
Decrease	*E. coli*	1% inulin/female	42	(84)
Decrease	*E. coli*	1% oligofructose/female	42	(84)
**Large intestine**				
Decrease	*Campylobacter*	1% inulin/female	42	(84)
Decrease	*Campylobacter*	1% oligofructose/female	42	(84)
Decrease	*E. coli*	1% inulin/female	42	(84)
Decrease	*E. coli*	1% oligofructose/female	42	(84)

However, adding high levels of fructans could result in negative impacts on broilers. Rapid fermentation by microbes in the intestine could produce too much SCFA, which damage intestinal mucosal barriers and increase intestinal permeability, consequently causing pathogen invasion, diarrhea, and poor growth performance (93, 94). Xu et al. (40) demonstrated that the addition of 0.2 or 0.4% of FOS in broiler diets could improve FCR and change cecal microbiota, but the supplementation of 0.8% of FOS had no significant differences compared with control treatment. It has been suggested that the supplementation of FOS above 0.5% is excessive; a previous report mentioned that birds fed with 0.5% FOS showed poorer growth performance and less intestinal *Lactobacillus* but higher titers of *E. coli* and *C. perfringens* compared with 0.25% FOS treatment (25). Furthermore, Biggs et al. (20) even showed that ME_n_ and amino acid digestibility were reduced by 8% short-chain FOS or inulin addition.

Fructans improved the immune responses of gut-associated lymphoid tissue (GALT) and the systemic immune system through three major mechanisms. Firstly, increasing the levels of *Bifidobacteria* by fructans could modulate the production of cytokines or antibodies. Secondly, leukocytes could be activated after their receptors respond to fructans' metabolites, such as SCFA. Thirdly, fructans could be directly recognized by carbohydrate receptors on the surface of immune cells (95). Huang et al. (39) reported that inulin reduced the levels of IL-6 and IFN-γ, increased IgA, and tended to increase the ratio of CD4^+^/CD8^+^ cells in the ileum of broilers. Moreover, Janardhana et al. (46) found that FOS could lead to systemic immune responses by increasing the levels of plasma antibody titers of IgG and IgM. Similarly, primary antibody titers against sheep red blood cells increased in broilers fed with FOS, but antibody titers in the secondary immune response were not influenced by FOS (59). Likewise, FOS increased IgA^+^ cells and upregulated TLR-4 and IFN-γ in the ileum of laying hens (90). Interestingly, there is a hypothesis that fructans might modulate the development of the immune system during embryogenesis. *In ovo* administration of inulin (d 12) downregulated the gene expression of IL-4, IL-12p40, IL-18, CD80, and interferon-β in the cecal tonsils of broilers on day 35 after hatching (60). Furthermore, *in ovo* injection of inulin had no adverse effect on GALT development but stimulated more colonization of lymphoid tissue by T cells in the cecal tonsil of broilers (96). To our knowledge, there are only a few studies that evaluated the *in ovo* administration of prebiotics. Further research is needed to understand what causes the different results between *in ovo* administration and direct-fed supplementation of fructans in broilers. It could be concluded that owing to the various fructans groups and DP, supplementation of fructans in diets might have affected broilers inconsistently. However, in a general review, fructans could modulate intestinal microorganisms, levels of intestinal SCFA, mucosal morphology, and generate immune responses.

## Other prebiotics

Besides the three major prebiotics, MOS, β-glucan, and fructans, other oligosaccharides have been evaluated and considered as potential prebiotics, including chitosan oligosaccharides (COS), galacto-oligosaccharides (GOS), galactoglucomannan oligosaccharide (GGMO), and xylo-oligosaccharides (XOS).

### Chitosan oligosaccharides (COS)

Extracted from chitin, COS contain 2–10 sugar units of *N*-acetyl glucosamine with 1–4 β-linkages. It has been reported that the supplementation of COS in broiler diets could modulate immune responses and enhance nutrient digestibility and feed efficiency. Huang et al. (97) indicated that chicken with COS supplementation had higher weight of bursa of Fabricius and thymus, higher IgG, IgA, and IgM in serum and higher antibody titers against Newcastle disease vaccines. On the other hand, 0.01% of COS improved ileal digestibility of dry matter, energy, crude protein, and most of the amino acids in broilers (21 or 42 d) (98). The improved digestibility of nutrients was associated with better growth performance in the same study (98). However, supplementation of COS above 0.01% might be excessive because chickens fed with 0.015% COS had significantly less body weight than birds fed with 0.01% COS (98).

### Galacto-oligosaccharides (GOS)

Galacto-oligosaccharides, synthetic prebiotics with galactose with 1–4 or 1–6 β-linkages, are normally produced from lactose by the enzyme lactase with high galactosyltransferase activity (99). *In ovo* injection of GOS could increase body weight of broilers 34 days after hatching (100). Administration of GOS also influenced the intestinal microbiota. Park et al. (85) reported that GOS treatment exhibited higher levels of *Alistipes* genus, *Lactobacillus intestinalis*, and *Faecalibacterium prausnitzii* in the ceca of broilers compared with the control group. Although Biggs et al. (20) demonstrated that GOS had no effects on cecal *Bifidobacteria* and *Lactobacillus* population, it has been reported that the addition of GOS in broiler diets could increase counts of *Bifidobacteria* in feces (101). Moreover, broilers that received *in ovo* GOS injection also had higher concentrations of *Bifidobacteria* and *Lactobacillus* in feces (102). The author suggested that *in ovo* administration of GOS could replace prolonged water supplementation. Owing to the inconsistent results, future studies are needed to confirm the effects of GOS in modulating intestinal microbial structures and further affecting immune responses in broilers.

### Galactoglucomannan oligosaccharides (GGMO) and galactoglucomannan oligosaccharides-arabinoxylan (GGMO-AX)

Galactoglucomannan oligosaccharides and galactoglucomannan oligosaccharides-arabinoxylan (GGMO-AX) are novel prebiotics extracted and processed from the wood chips of softwood trees (103). These oligosaccharides consist of mannose, glucose, and galactose monomers. An *in vitro* investigation showed that *Lactobacillus* could grow faster on GGMO than MOS (35). The same research also indicated that the supplementation of 0.2% GGMO in broiler diets could reduce colonization of *Salmonella typhimurium* in the ileum, ceca, and liver; as a consequence of clearing *S. typhimurium* infection, GGMO ameliorates intestinal morphology and growth performance compared with a *Salmonella*-challenged control treatment (35). The improvement might be attributed to the modulation of immune responses by GGMO. Faber et al. (104) reported that the *Eimeria acervulina-*challenged birds that received 4% GGMO-AX showed enhanced gene expression of pro-inflammatory cytokines, including IFN-γ, IL-1β, IL-6, and IL-12β, but also showed decreased levels of anti-inflammatory cytokines such as interleukin-15. Galactoglucomannan oligosaccharides-arabinoxylan might not only affect immune responses in broilers but also alter intestinal microbial population. It has been shown that the administration of 2% GGMO-AX increased counts of *Bifidobactrium* spp. in the ceca (104) and 4% GGMO-AX decreased the concentration of *C. perfringens* (105). Although the supplementation of GGMO-AX in high levels showed some positive effects on broilers, simultaneously, it could lead to poor growth performance (104). Therefore, further studies should evaluate the administration of GGMO or GGMO-AX in appropriate concentration to maintain growth performance and improve the health status of broilers at the same time.

### Xylo-oligosaccharides (XOS)

Xylo-oligosaccharides are oligosaccharides, which consist of xylose sugar units with β-linkages (42). Xylan, the main component of cereal fiber such as corn cobs, straws, hulls, and bran are the raw resources for XOS production (106). Xylan could be degraded to XOS by xylanase of fungi, steam, or diluted solutions of mineral acid (106). Similar to other prebiotics, XOS could improve growth performance, increase the intestinal villus height, increase the proportion of *Lactobacillus*, and enhance the levels of acetate, butyrate, and lactate in the ceca of broilers (42, 107, 108). It was suggested that XOS would improve humoral immunity in poultry. An increase in antibody titers against avian influenza H5N1 was observed in broilers by XOS addition (107). Furthermore, De Maesschalck et al. (42) speculated that XOS could lead to cross-feeding mechanisms between *L. crispatus* and *Anaerostipes butyraticus* in the gut of the broiler. Owing to XOS fermentation, *L. crispatus* produces lactate, which might be utilized by butyrate-producing bacteria that belong to members of *Clostridium* cluster XIVa. This hypothesis was further supported by the observation of increasing numbers of cecal *Clostridium* cluster XIVa and butyryl-CoA: acetate-CoA transferase, a marker indicating the butyrate-producing capacity of intestinal microbiota (42). As mentioned above, butyrate is a major energy source for intestinal epithelial cells. Apart from acting as an important fuel in the intestine, butyrate can stimulate *MUC-2* gene expression, exert anti-inflammatory effects, and prevent necrotic enteritis from pathogenic infection (109–111). In summary, XOS supplementation would enhance cross-feeding mechanisms and produce butyrate, consequently leading to beneficial influences on broilers.

### *In ovo* injection

Direct feeding and *in ovo* injection are two main strategies for applying prebiotics. Prebiotic can be administrated by injecting 0.2 ml aqueous solution into the air chamber of eggs on day 12 of embryonic incubation (112). *In ovo* injection of prebiotics can alter microbial community in embryonic guts, improve intestinal morphology, and directly promote robustness of both cellular and humoral immune responses in the GALTs of the neonate post hatching (96, 113, 114).

The embryonic microbiota is different from the intestinal microbiota of post hatching and adult birds. The dominant bacterial phylum is *Proteobacteria*, followed by *Firmicutes, Bacteroidetes*, and *Actinobacteria* in the chicken embryos (115). In addition, the embryonic microbial community is altered during the development of the embryos. The 19-day-old embryos exhibited more microbial diversity than the 4-day-old embryos. The proportion of *Proteobacteria* decreased, whereas *Firmicutes, Bacteroidetes*, and *Actinobacteria* increased in the 19-day-old embryos compared with the 4-day-old embryos (115). Even though *Proteobacteria* decreased in the late embryonic development period, this phylum dominated in early-age birds until *Firmicutes* became prominent after 7 days post hatching (116). However, the embryonic microbiota could be contaminated by pathogens directly from the yolk, yolk membranes, albumen, shell membranes originating from the reproductive organs of laying hens, or indirectly from the egg shells. Pathogens such as *Salmonella* located in the albumen were able to migrate and penetrate the vitelline membrane and grow in the yolk (117). On the other hand, it was suggested that spore forming bacteria such as *Clostridium tertium* were capable of surviving the disinfection process and penetrating eggs, resulting in contamination (118). To avoid extensive pathogen infection, prebiotics were delivered *in ovo*, which is likely fermented by the indigenous embryonic microbiota, inhibiting pathogen proliferation and regulating gene expression of immune responses (119). Villaluenga et al. (120) reported that injection of raffinose at day 12 of embryonic incubation had the highest amounts of *Bifidobacteria* in the ceca of 2 day-old broilers. Additionally, they indicated that 8.815 mg per egg of raffinose delivered *in ovo* reduced embryo weight. A later research showed that 4.5 mg of raffinose that was delivered *in ovo* had no significant effects on body weight but enhanced gene expression of *CD3* and *ChB6*, which are associated with the activity of T cells and B cells (114). Moreover, villus height and villus height to crypt depth ratio of post hatching birds increased linearly with higher dosages of raffinose (114). *In ovo* injection of inulin and GOS also increased villus height in the jejunum of 1-day-old chickens (92). Moreover, administration of GOS *in ovo* showed differential gene expression in the ceca related to lymphocyte proliferation, activation, and differentiation and cytokine production (119). This study pointed out that *GZMA* (Granzyme A), a cytotoxic T cell-specific gene, was upregulated in the cecal tonsil of birds delivered with GOS *in ovo*. Similarly, other research has also demonstrated that GOS increased helper T cells in the cecal tonsil and B cells in the bursa of Fabricius (96). Furthermore, beta inhibin and lectin galactoside-binding soluble 3, which are related to regulation of T cell and innate immunity, were upregulated by GOS. On the other hand, GOS also downregulated the *SERPING1* gene, which could inhibit part of the complement cascade system (119). It was suggested that the *in ovo* injection of GOS might not only regulate intestinal innate and adaptive immune system but also modulate gene expression of nutrient digestion and transportation. Firstly, chicken injected with GOS *in ovo* exhibited higher levels of sodium-dependent glucose co-transporters in the intestine, which are related to the absorption of monosaccharides (119). Secondly, birds delivered with GOS *in ovo* showed increased amylase and trypsin activity of the pancreas on embryonic day 21 and day 7 post hatching respectively (100). These studies led us to a conclusion that *in ovo* injection of prebiotics could affect the ecosystem of broilers, but, to our knowledge, little research has compared the difference between the direct-fed method and *in ovo* injection. A study reported that injection of galacto-oligosaccharides into eggs could increase *Bifidobacteria* and *Lactobacillus* in the feces of broilers. Though the author suggested that *in ovo* injection could replace prolonged supplementation via water system (102), more studies are needed to compare these two different approaches on the application of prebiotics.

## Conclusion

The interaction between epithelium, microbiota, and immunity in animal gut is complicated. Recent data have demonstrated that prebiotics potentially alter the interaction between the host and gut microbiota and improve the health status of broilers. However, the interaction is sometimes induced by certain prebiotics or host species. Therefore, it is inevitable that prebiotics showed variable effects on animals. Still, most prebiotics can be fermented by beneficial bacteria, and the increased levels of *Lactobacillus* and *Bifidobacteria* or their metabolites may inhibit pathogen colonization and communicate with epithelial cells and immune cells. By improving gut environment or immune responses, prebiotics further provide resistance to pathogens and maintain efficient production. In addition, some prebiotics can be recognized by sentinel cells directly, triggering cytokines' cascade, which results in the upregulation of innate or humoral immunity. Although previous studies have discovered some mechanisms that participate in the cross talk between prebiotics and the ecosystem of the gut, there are still several hypotheses, which shall be confirmed in the future. In this context, administration of prebiotics presents tremendous influences on the broilers' gut health by the modulation of the gut microbial community and the interaction between the host immune system and gut microbiota. It is suggested that prebiotics delivered *in ovo* or fed directly can act as alternatives to antibiotics because of the significant improvement of microbial community, intestinal integrity, and immunity of the host.

## Author contributions

P-YT reviewed papers related to the topics and wrote the manuscript. WK reviewed papers related to the topic, gave directions and ideas to P-YT, and reviewed and revised the manuscript.

### Conflict of interest statement

The authors declare that the research was conducted in the absence of any commercial or financial relationships that could be construed as a potential conflict of interest.
